# Larval metamorphosis is inhibited by methimazole and propylthiouracil that reveals possible hormonal action in the mussel *Mytilus coruscus*

**DOI:** 10.1038/s41598-021-98930-9

**Published:** 2021-09-29

**Authors:** Yi-Feng Li, Yu-Qing Wang, Yi Zheng, Xue Shi, Chong Wang, Yu-Lan Cheng, Xin Zhu, Jin-Long Yang, Xiao Liang

**Affiliations:** 1 International Research Center for Marine Biosciences, Ministry of Science and Technology, Shanghai Ocean University, Shanghai, China; 2 Key Laboratory of Exploration and Utilization of Aquatic Genetic Resources, Ministry of Education, Shanghai Ocean University, Shanghai, China; 3 Ocean and Fisheries Research Institute of Binzhou, Binzhou, China; 4 College of Fisheries and Life Science, Shanghai Ocean University, 999 Hucheng Huan Road, Shanghai, 201306 China

**Keywords:** Animal physiology, Morphogenesis

## Abstract

Larval metamorphosis in bivalves is a key event for the larva-to-juvenile transformation. Previously we have identified a thyroid hormone receptor (TR) gene that is crucial for larvae to acquire “competence” for the metamorphic transition in the mussel *Mytilus courscus* (Mc). The mechanisms of thyroid signaling in bivalves are still largely unknown. In the present study, we molecularly characterized the full-length of two iodothyronine deiodinase genes (McDx and McDy). Phylogenetic analysis revealed that deiodinases of molluscs (McDy, CgDx and CgDy) and vertebrates (D2 and D3) shared a node representing an immediate common ancestor, which resembled vertebrates D1 and might suggest that McDy acquired specialized function from vertebrates D1. Anti-thyroid compounds, methimazole (MMI) and propylthiouracil (PTU), were used to investigate their effects on larval metamorphosis and juvenile development in *M. coruscus*. Both MMI and PTU significantly reduced larval metamorphosis in response to the metamorphosis inducer epinephrine. MMI led to shell growth retardation in a concentration-dependent manner in juveniles of *M. coruscus* after 4 weeks of exposure, whereas PTU had no effect on juvenile growth. It is hypothesized that exposure to MMI and PTU reduced the ability of pediveliger larvae for the metamorphic transition to respond to the inducer. The effect of MMI and PTU on larval metamorphosis and development is most likely through a hormonal signal in the mussel *M. coruscus*, with the implications for exploring the origins and evolution of metamorphosis.

## Introduction

Metamorphosis encompasses a spectacular post-natal developmental process involving a series of morphological and physiological changes that enable larva to adult transformation occurring from invertebrates to vertebrates^1–3^. In metamorphosing anurans, the remodeling of internal organs, the tail's resorption, and the limbs' appearance are dependent on thyroid hormones (THs) by binding to thyroid hormone receptors and initiating downstream cellular responses^2^. In teleost flatfish, THs are essential factors for the symmetric pelagic larva to transform into an asymmetric benthic adult^4^. In urochordates, THs have been found in ascidian *Ciona intestinalis* larvae and its involvement in larval metamorphosis^5^, whereas in cephalochordates, triiodothyroacetic acid (TRIAC) as a THs derivative, rather than T3 and T4, trigger metamorphosis in the amphioxus^3^. Endogenous THs synthesis has been reported in molluscs such as sea hare *Aplysia californica* and Pacific oyster *Crassostrea gigas*^6,7^. Exogenous THs exposure induced larval metamorphosis of two abalone species *Haliotis gigantea* and *H. discus discus*^8^. The identification of iodothyronine deiodinase genes in several non-chordate species suggests that THs metabolism may also exist in invertebrates^9,10^.

Iodothyronine deiodinases are enzymes that can activate and inactivate THs molecules and play an essential role in modulating THs metabolism in the body^11^. THs are synthesized in the thyroid gland and secreted to serum, mainly thyroxine (T4; 3,5,3′,5′-tetraiodothyronine)^12^. Three different types of iodothyronine deiodinases are present in vertebrates, type 1 (D1), type 2 (D2) and type 3 (D3). The conversion of THs into active or inactive hormones was dependent on the ring-specific iodine removal. 3,5,3′-Triiodothyronine (T3), an active form of THs, is generated by removing outer-ring iodine from T4 molecules catalyzed by D1 and D2^13^. D1 and D3 inactivate T3 by inner-ring deiodination^13^. Despite differences in the function of selectively eliminating iodine moieties from two phenyl rings in iodothyronines, the removal of iodide in three enzymes is both dependent on the rare amino acid selenocysteine (Sec) in their core active center^14^. The core active center is highly conserved among almost all types of deiodinases in various species consisting of approximately 15 amino acid residues^15,16^. Sec residue is encoded by UGA that generally translates as a stop codon. The presence of a selenocysteine insertion sequence (SECIS) element in the 3′-UTR of mRNA is essential for the synthesis of deiodinases by recoding UGA to Sec codon insertion rather than translation termination^17^.

Anti-thyroid compounds such as 6-*n*-propyl-2-thiouracil (PTU) and methimazole (MMI) have been employed to disrupt the thyroid axis for investigating the effect of THs synthesis and metabolism in larval development and growth of vertebrates^18,19^. PTU selectively inhibits deiodinase activity for blocking T3 production, particularly D1^12^. However, not all vertebrates D1 are inhibited by PTU. Unlike mammals D1 with highly sensitivity to PTU, D1 in *Xenopus laevis* and tilapia (*Oreochromis niloticus*) were PTU-insensitive enzymes^20,21^. Site-directed mutagenesis in *Xenopus* demonstrated that the difference of D1 sensitivity to PTU was dependent on a specific amino acid in the core catalytic center of D1^21^. Methimazole (MMI) has an inhibitory effect on thyroid hormone biosynthesis by interfering with thyroid peroxidase-mediated iodination of thyroglobulin in the thyroid gland^22^. Marine phytoplankton such as microalgae is a rich source of iodine in the ocean^23^, which is consumed by filter-feeding bivalves. The presence of putative deiodinases and thyroid peroxidase genes in *M. coruscus* transcriptome leads us to speculate that accumulated iodine in mussels could be used to iodinate their proteins which may be important for growth and development.

Previously we identified a thyroid hormone receptor (TR) gene in *Mytilus coruscus* (Mc), and knock-down of TR gene affected epinephrine-induced larval metamorphosis^24^. It led us to hypothesize that the TR gene affected the pediveliger acquiring “competence” for metamorphic transition in *M. coruscus*^24^. The presence of putative deiodinases and TR orthologs in some molluscs and annelids may suggest a regulatory role in development^7,9–11,25^. Comparative researches of TH signaling in invertebrates may contribute to a better understanding of this ancient origin. Here, we cloned and characterized the full-length of two deiodinase genes (McDx and McDy) in *M. coruscus*. Phylogenetic analysis revealed that McDy clustered with two deiodinase genes from Pacific oyster *C. gigas* (CgDx and CgDy) and shared a common origin with vertebrates (D2 and D3). MMI and PTU, as iodination and deiodination of proteins inhibitors, were employed to investigate the effects of iodinated proteins on larval metamorphosis and juvenile growth of the mussel *M. coruscus*.

## Materials and methods

### *M. coruscus* larvae and juvenile rearing

The spawning and larvae culture method of *M. coruscus* was performed as previously described^24^. In summary, adult mussels were induced for spawning by exposure to air. Fertilization was performed by gently mixing sperms and eggs in filtered seawater (FSW; acetate-fibre filter: 1.2 μm pore size) at 18 °C. Fertilized eggs were kept in clean FSW for 2 days at 18 °C after removing the excess sperms through nylon plankton net (mesh size: 20 μm). Larvae were maintained at a density of 5 larvae mL^−1^ at 18 °C until they reached the pediveliger stage when metamorphosis bioassays were performed. The microalgae *Isochrysis zhanjiangensis* was provided as a food resource. Juveniles (post-metamorphic) were obtained and cultured for determining the effect of 4 weeks’ exposure to MMI and PTU on juvenile growth. All procedures for mussel acclimation and experimentation were authorized by the Animal Ethics committee of Shanghai Ocean University with the registration number of SHOU-DW-2018-013.

### Total RNA extraction and cDNA synthesis

Total RNA was isolated with the RNAiso Plus reagent according to the manufacturer’s instructions (Takara, Japan) and genomic DNA was eliminated by DNAse (Ambion Turbo DNase kit). The quality of DNase treated total RNA samples was determined by 2100 Bioanalyser (Agilent Technologies, Inc., Santa Clara CA, USA) and quantified using the ND-2000 (NanoDrop Thermo Scientific, Wilmington, DE, USA). cDNA synthesis was carried out using the PrimeScript Reagent Kit (Takara, Japan) with DNase-treated RNA.

### Molecular cloning and characterization of the full-length of two *M. coruscus* deiodinase sequences

The full-length of two *M. coruscus* deiodinase genes were amplified using a SMARTer™ RACE 5′/3′ Kit (Clontech, Japan). Primers for 3′ and 5′ RACE were designed according to the partial deiodinase transcript sequences based on *M. coruscus* transcriptome (unpublished data, Table 1). Touchdown PCR with the universal primer A mix (UPM primer) and a gene-specific primer was carried out using the following PCR program: 94 °C for 4 min, 5 cycles of 94 °C for 30 s, 72 °C for 2 min, and another 5 cycles of 94 °C for 30 s, 70 °C for 30 s, and 72 °C for 2 min, followed by another 35 cycles of 94 °C for 30 s, 68 °C for 30 s, and 72 °C for 2 min. The final elongation step was performed for 10 min at 72 °C. The second round PCR with the universal primer short (UPM short) and gene-specific primers were performed according to the following PCR program: 94 °C for 4 min, 35 cycles of 94 °C for 30 s, 63 °C for 30 s, and 72 °C for 90 s, and a final extension at 72 °C for 10 min. The PCR products were isolated, purified and cloned into a pMD 19-T vector (TaKaRa, Dalian, China) and confirmed by sequencing (Sangon Biotech, China).

**Table 1:** List of the primers used in RACE PCR and real-time quantitative PCR (qPCR) in this study.

Primer	Sequence (5′–3′)	Amplicon size (bp)	*R*^2^	Efficiency (%)	Application
McDx-5’R1	ACATCACAATAAAACCACCAACACT				5′RACE
McDx-5’R2	TGATGCCCTGGCCTCTAAGTATTCC				5′RACE
McDx-3’F1	GCAGACGAATGGAGTGTACAAGGCA				3′RACE
McDx-3’F2	AGTGCTTTCCCAGAACGTTACTACG				3′RACE
McDy-3’F1	CAGCAGCAAAACGGCTACAATCTT				3′RACE
McDy-3’F2	GGTGAAAGAGGACCAGTAGGGTATCG				3′RACE
McDx-F	AGCTGCTCTTGACCACCGTTTATG	142	0.99	96	qPCR
McDx-R	ACGTTGTGCCTTGTACACTCCATTC				qPCR
McDy-F	TTCAAACAAGTATGGAGGGCGATGT	179	0.99	98	qPCR
McDy-R	AACGGCGGTCACGAACAACTG				qPCR

The Maximum Likelihood (ML) tree was built in PhyML 3.0^26^ available at the ATGC bioinformatics platform using the JTT substitution model obtained by an SMS automatic model selection^27^. The reliability of internal branching was performed with 100 bootstrap replicates. Human glutathione peroxidase (HsGPx, CAA41228) served as an outgroup. The SECIS elements of two deiodinase genes in the 3′-UTRs of *M. coruscus* were generated by the online tool SECISearch3 (http://seblastian.crg.es)^28^.

### Quantitative real-time PCR analysis

McDx and McDy transcripts in five developmental stages (trochophore, D-veliger, umbo, pediveliger and post-larvae; Fig. S1) were determined by quantitative real-time PCR analysis (qPCR). Specific primers for McDx and McDy are listed in Table 1. qPCR analysis of McDx and McDy was conducted with five biological replicates for each developmental stage in 96 multi-well plates using a LightCycler 960 (Roche). An absolute quantification method was performed as previously described^24^. Quantification was conducted using the standard curve method with the template isolated from PCR bands (the standards), which were sequenced to confirm their identity prior to qPCR reactions. A standard curve with 10^7^–10^1^ DNA copies of the target amplicon was included in each qPCR reaction. The copy number of the target gene based on the threshold cycle (CT) value of each sample was calculated according to the standard curve. qPCR reactions were performed in duplicate, and each reaction containing 1 μL template cDNA, 0.3 μL of each forward and reverse primers (10 mM), 5 μL of 2× FastStart Essential DNA Green Master (Roche) and sigma water to give a final reaction volume of 10 μL. qPCR amplification protocol was carried out as follows: an initial denaturation step at 95 °C for 10 min followed by 45 cycles of 10 s at 95 °C and 10 s at the optimal annealing temperature for specific primers. A melting curve analysis was carried out to confirm that a single amplified product was obtained.

### MMI and PTU blocking of larval metamorphosis

The effect of MMI and PTU on larval metamorphosis was carried out as previously described with some modifications^29^. Briefly, pediveliger larvae were exposed to the desired concentration of MMI and PTU for 24 h. Pediveliger larvae were rinsed three times with autoclaved filtered seawater (AFSW) prior to epinephrine (EPI) exposure. EPI, an active inducer, was used for inducing larval metamorphosis of *M. coruscus*^24^. After exposure to MMI and PTU, twenty pediveliger larvae were exposed to 10^–4^ M EPI for 96 h in each Petri dish (Ø 64 × 19 mm height). The chemical solution was not changed during the experiment. Six replicates of each treatment group were carried out. The treatment groups that were only treated with MMI or PTU without the addition of EPI were performed. The pediveliger larvae only exposed to AFSW alone were set up as blank control. The pediveliger larvae treated with 10^–4^ M EPI were selected as a positive control (PC).

Juvenile mussels were exposed to MMI or PTU at concentrations ranging from 10^–3^ to 10^–5^ M for 4 weeks to evaluate their effect on the growth of *M. coruscus*. Juveniles were incubated in Petri dishes containing twenty juveniles and each test chemical solution. Shell length and dead juveniles were determined every other day. For each treatment group, shell length growth was calculated from 50 juveniles and viability was recorded with 12 biological replicates. Chemical solutions were renewed every other day. Juveniles were fed with 1 × 10^5^ cells ml^–1^ day^–1^ *I. zhanjiangensis* during the culture period. All bioassays were performed in a dark environment at 18 °C.

### Statistical analysis

Data were analysed using JMP™ software. The percentage of post-larvae (larval metamorphosis data) was arcsine-transformed and tested for normality (Shapiro–Wilk test) and homogeneity (O'Brien test). A *p*-value < 0.05 was the cut-off for statistic difference. Metamorphosis data was analysed using Wilcoxon/Kruskal Wallis test.

## Results

### Cloning, evolutionary and qPCR analysis of two deiodinase genes in *M. coruscus*

**Figure 1:**
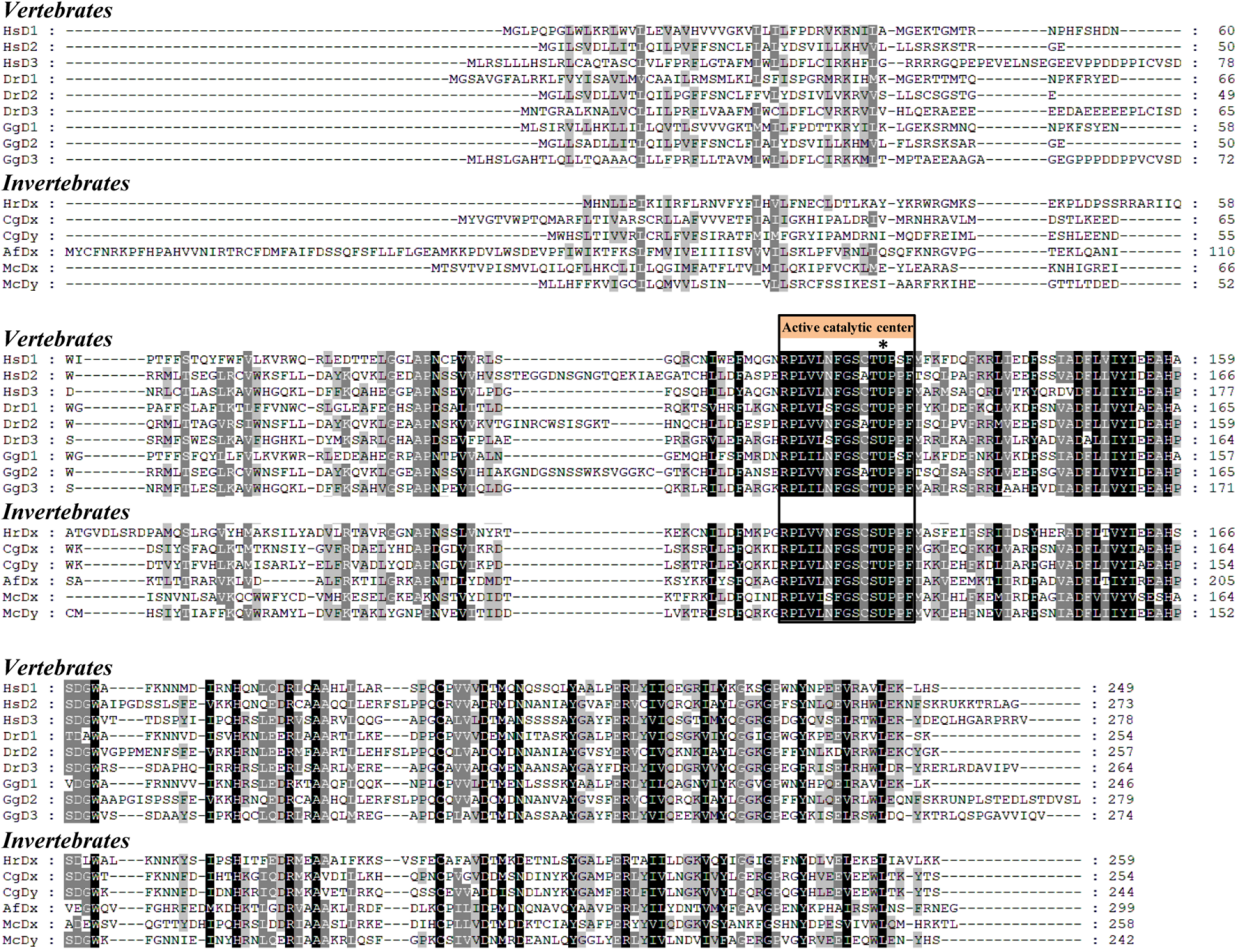
Multiple sequence alignment of two *M. coruscus* deiodinases with the deiodinases from vertebrates and invertebrates. The conserved active catalytic centers of the deduced amino acid sequences are indicated in orange. Identical amino acids are presented in black, similar amino acids in grey. An asterisk indicates a selenocysteine. HsD1 (*Homo sapiens*, AAB23670), HsD2 (*H. sapiens*, AAD45494), HsD3 (*H. sapiens*, AAH17717), DrD1 (*Danio rerio*, NP_001007284), DrD2 (*D. rerio*, NP_997954), DrD3 (*D. rerio*, NP_001242932), GgD1 (*G. gallus*, NP_001091083), GgD2 (*G. gallus*, AAD33251), GgD3 (*G. gallus*, NP_001116120), HrDx (*Halocynthia roretzi*, AAR25890), CgDx (*C. gigas* AKF17655), CgDy (*C. gigas* AKF17656), AfDx (*A. farreri*, AEX08671), McDx (*M. coruscus*, MW928627) and McDy (*M. coruscus*, MW928628).

**Figure 2:**
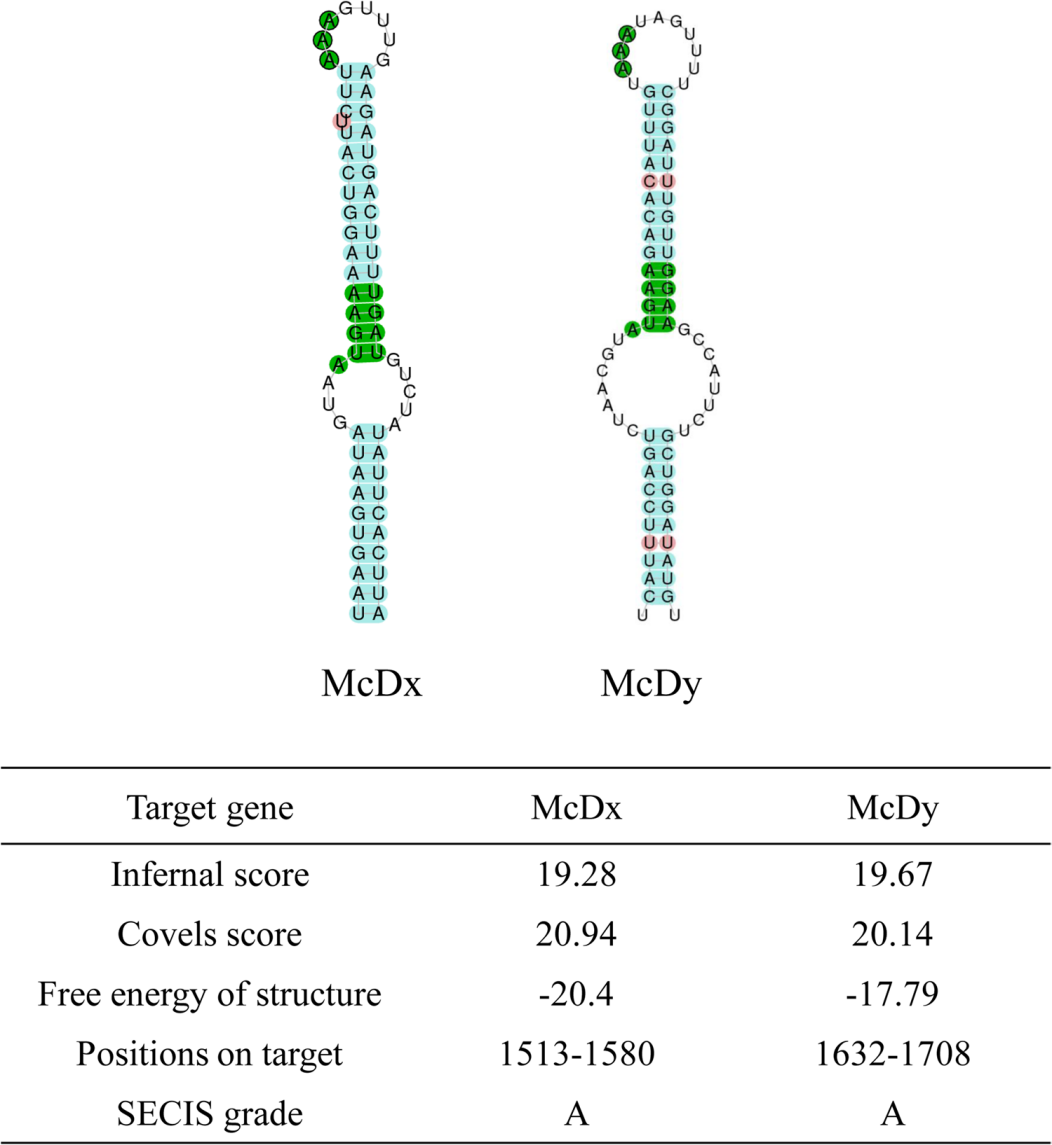
The SECIS elements of two deiodinase genes of *M. coruscus* were generated by SECISearch3 (Mariotti et al. 2013). The core and the unpaired conserved nucleotides are presented in green, and mismatches are indicated in red.

**Figure 3:**
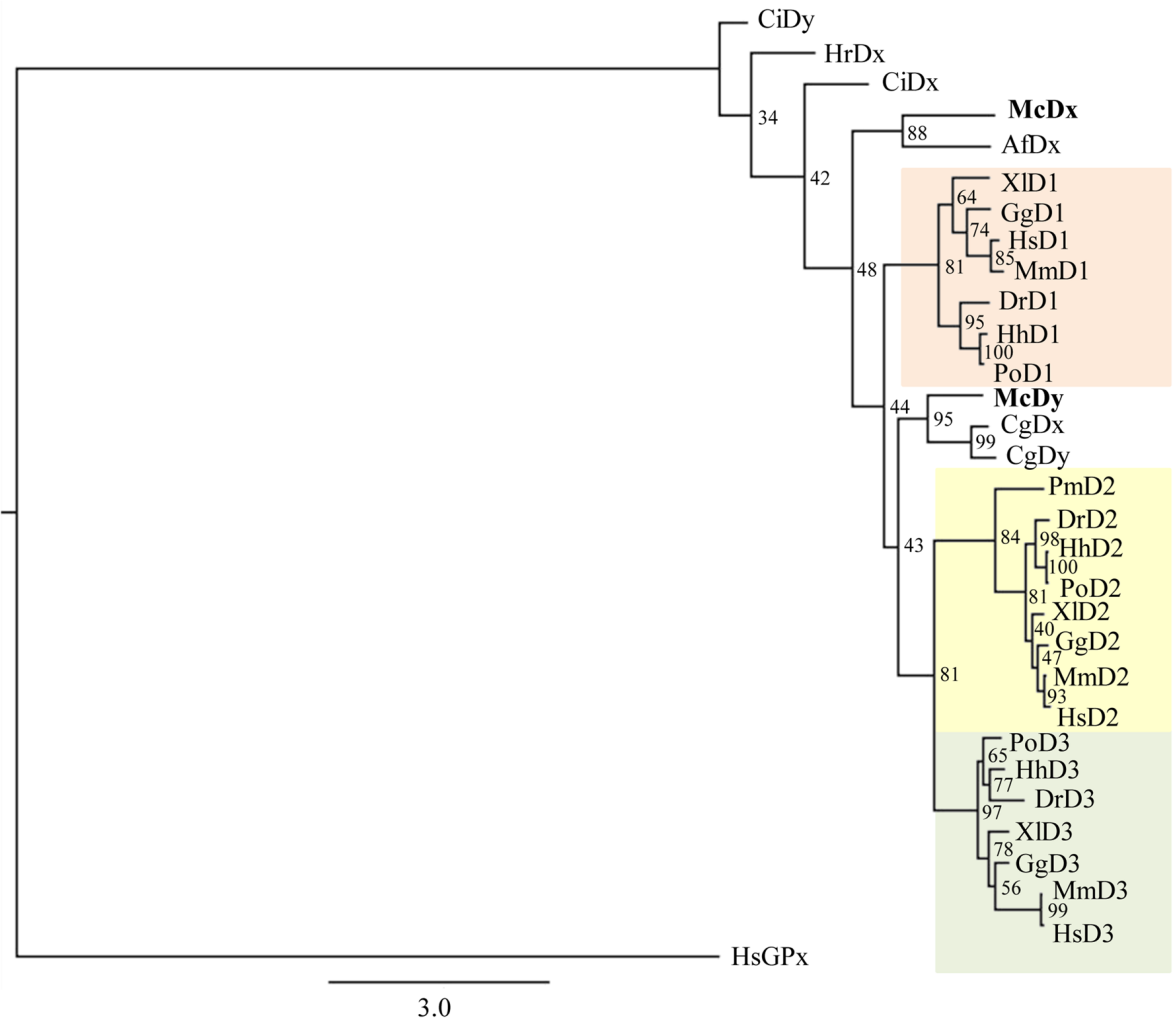
Phylogenetic analysis of two *M. coruscus* deiodinases with other metazoan homologues. The tree was built using the maximum-likelihood (ML) method based on the full-length amino acid sequence of deiodinases. The chordate D1 and a cluster include McD1, AfDx and SpD1 were boxed in pink, the chordate D2 in yellow and the chordate D3 in green. The sequence of two *M. coruscus* deiodinases are highlighted in bold. The tree was rooted with the sea anemone iodotyrosine deiodinase 1 (IYD1, XP_001633169). HsD1 (*H. sapiens*, AAB23670), HsD2 (*H. sapiens*, AAD45494), HsD3 (*H. sapiens*, AAH17717), MmD1 (*Mus musculus*, NP_031886), MmD2 (*M. musculus*, NP_034180), MmD3 (*M. musculus*, AAI06849), GgD1 (*G. gallus*, NP_001091083), GgD2 (*G. gallus*, AAD33251), GgD3 (*G. gallus*, NP_001116120), XlD1 (*X. laevis*, AAZ43088), XlD2 (*X. laevis*, AAK40121), XlD3 (*X. laevis*, AAA49971), DrD1 (*D. rerio*, NP_001007284), DrD2 (*D. rerio*, NP_997954), DrD3 (*D. rerio*, NP_001242932), PoD1 (*Paralichthys olivaceus*, BAG15906), PoD2 (*P. olivaceus*, BAG15907), PoD3 (*P. olivaceus*, BAG15908), HhD1 (*Hippoglossus hippoglossus*, ABI93488), HhD2 (*H. hippoglossus*, ABI93490), HhD3 (*H. hippoglossus*, ABI93489), PmD2 (*Petromyzon marinus*, KC306946), HrDx (*H. roretzi*, AAR25890), CiDx (*C. intestinali*; XP_026689666); CiDy (*C. intestinali*; XP_009859641); CgDx (*C. gigas* AKF17655), CgDy (*C. gigas* AKF17656), AfDx (*A. farreri*, AEX08671), McDx (*M. coruscus*, MW928627) and McDy (*M. coruscus*, MW928628).

Full-length of two deiodinase genes (McDx and McDy) were cloned using RACE PCR in *M. coruscus*. The full-length cDNA sequences of McDx (Genbank accession number MW928628) and McDy (Genbank accession number MW928627) were 1773 and 1755 nucleotides coding for a protein of 258 and 242 amino acids, respectively (Fig. 1). Both McDx and McDy contain a TGA codon recoding Sec residue in the active catalytic center, which showed high similarity with the vertebrate and other invertebrate deiodinases. The SECIS elements in the 3′-UTRs of McDx and McDy were identified by SECISearch3 (https://seblastian.crg.es/), and the core bears the conserved sequence UGAN/KGAW with the non-canonical pairing of AG-GA (Fig. 2)^28^. The conserved adenines in the apical loop and SECIS grade of two deiodinases were predicted as A, which proved that McDx and McDy were selenoproteins. ML phylogenetic tree based on the full-length of McDx and McDy amino acids sequences was constructed to investigate the evolutionary relationship (Fig. 3). Vertebrates D2 and D3 were closely clustered and two deiodinases responsible for the outer-ring deiodination and inner-ring deiodination, respectively. McDy shared high similarity with two deiodinases of oyster *C. gigas* (CgDx and CgDy) and formed a cluster grouping with a clade of D2 and D3 from vertebrates (Fig. 3). It may suggest that the function of McDy is closer to D2 and D3 from the evolutionary perspective. ML tree showed that McDx was grouped with scallop *Azumapecten farreri* (AfDx) and clustered outside of three types of vertebrate deiodinase groups (Fig. 3).

**Figure 4:**
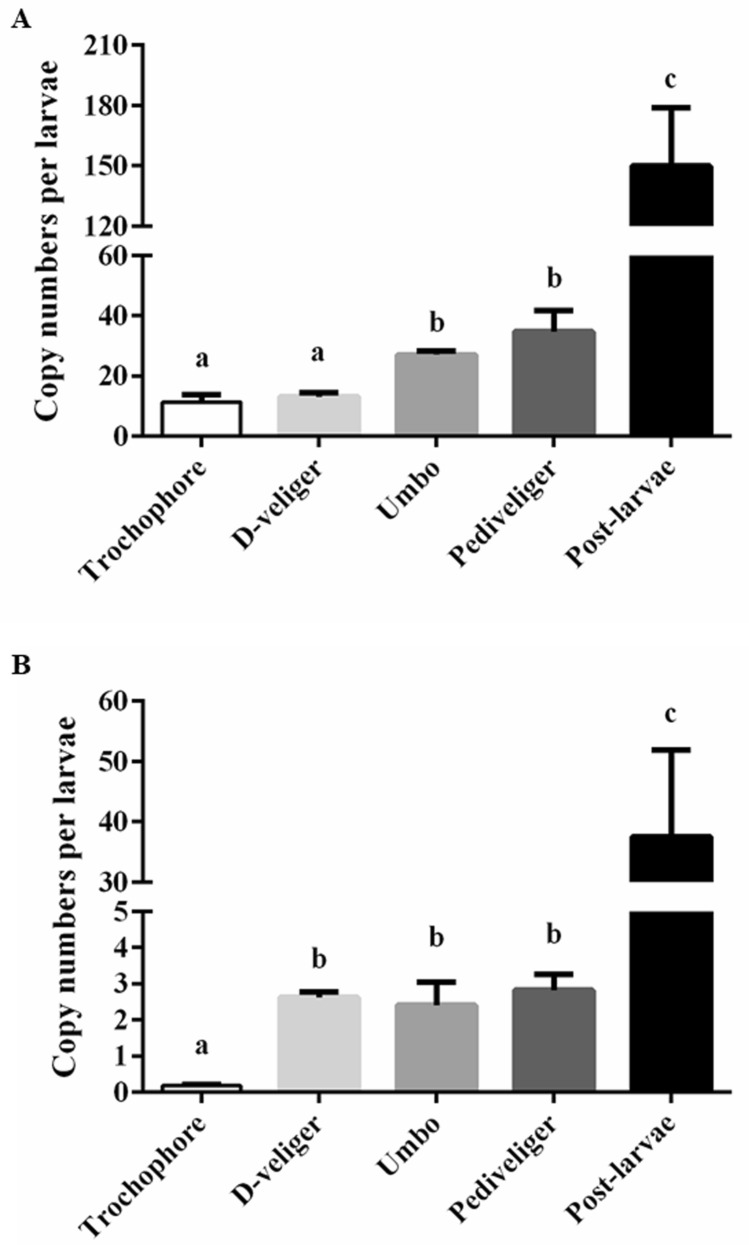
McDx (**A**) and McDy (**B**) transcript abundances in five developmental stages (trochophore, D-veliger, umbo, pediveliger and post-larvae) of *M. coruscus*. Different letters indicate significant difference (*P* < 0.05).

The transcriptional expression of McDx was significantly upregulated in umbo and pediveliger stage relative to two early larval stages (trochophore and D-veliger) (*P* < 0.05; Fig. 4A). The mRNA of both McDx and McDy were significantly higher in the post-larvae stage compared to the other stages (*P* < 0.05; Fig. 4).

### Effect of MMI and PTU on larval metamorphosis and growth of *M. coruscus*

**Figure 5:**
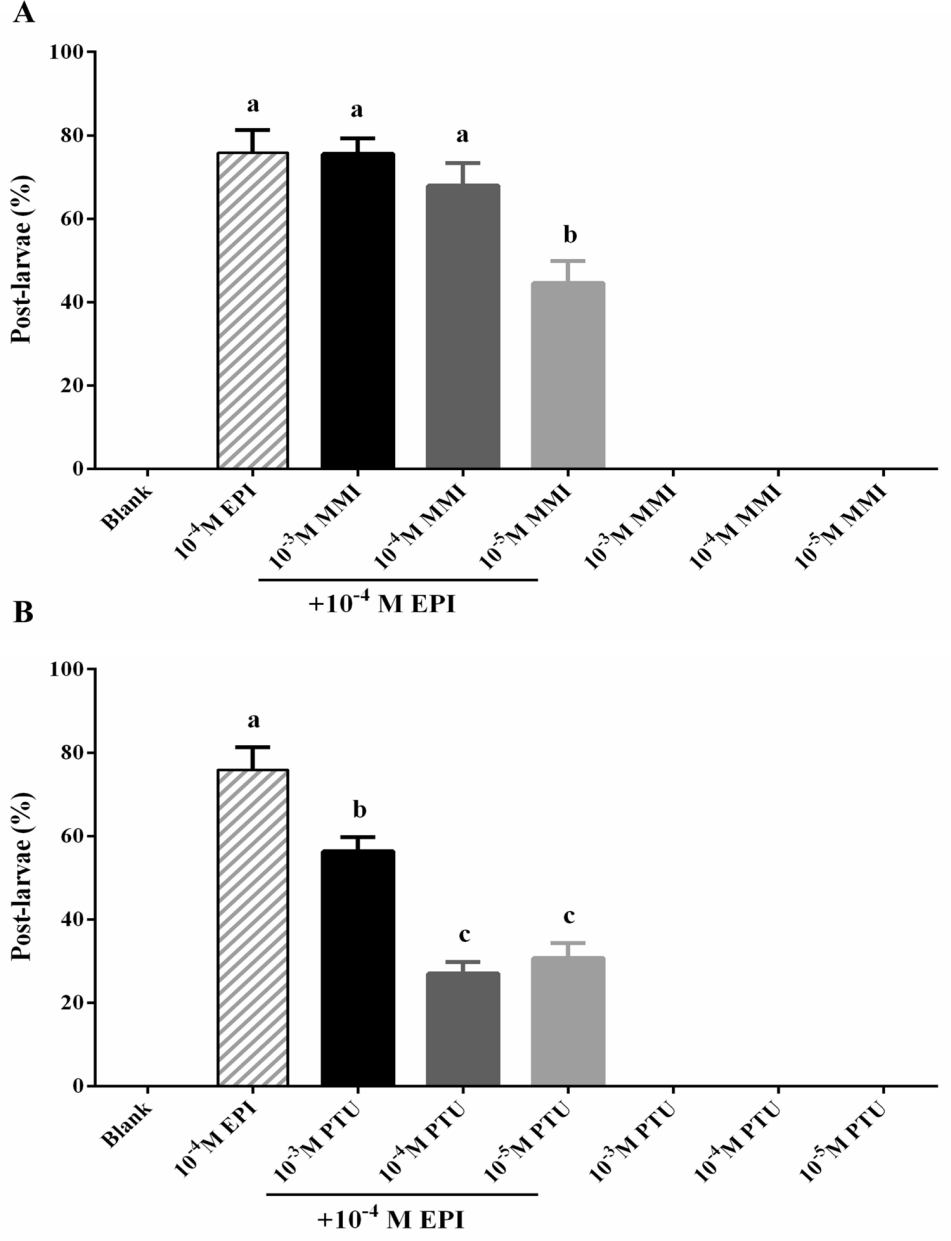
Effect of MMI (**A**) and PTU (**B**) on larval metamorphosis of *M. coruscus* at 96 h in the presence or absence of 10^–4^ M EPI. Data are represented as mean ± SEM of six biological replicates and each containing 20 larvae per replicate. Different letters indicate significant difference (*P* < 0.05).

The effect of MMI and PTU on larval metamorphosis in the presence/absence of 10^–4^ M EPI is shown in Fig. 5. MMI or PTU alone did not promote larval metamorphosis without the addition of 10^–4^ M EPI (Fig. 5). 76% of the pediveliger larvae exposed to 10^–4^ M EPI underwent metamorphosis (Positive control; PC) (Fig. 5A). Treatment with 10^–5^ M MMI significantly inhibited larval metamorphosis compared to the PC group (*P* < 0.05), while 10^–3^ and 10^–4^ M MMI had no effect on larval metamorphosis relative to the PC group (*P* > 0.05) (Fig. 5A). PTU significantly inhibited larval metamorphosis in all three tested concentrations relative to the PC group (*P* < 0.05), and two lower concentrations of PTU (10^–4^ M and 10^–5^ M) had a pronounced inhibition effect than a higher concentration (10^–3^ M) (*P* < 0.05) (Fig. 5B). Larval viability was not affected after exposure to EPI for 96 h (*P* > 0.05) (Fig. S2).

**Figure 6:**
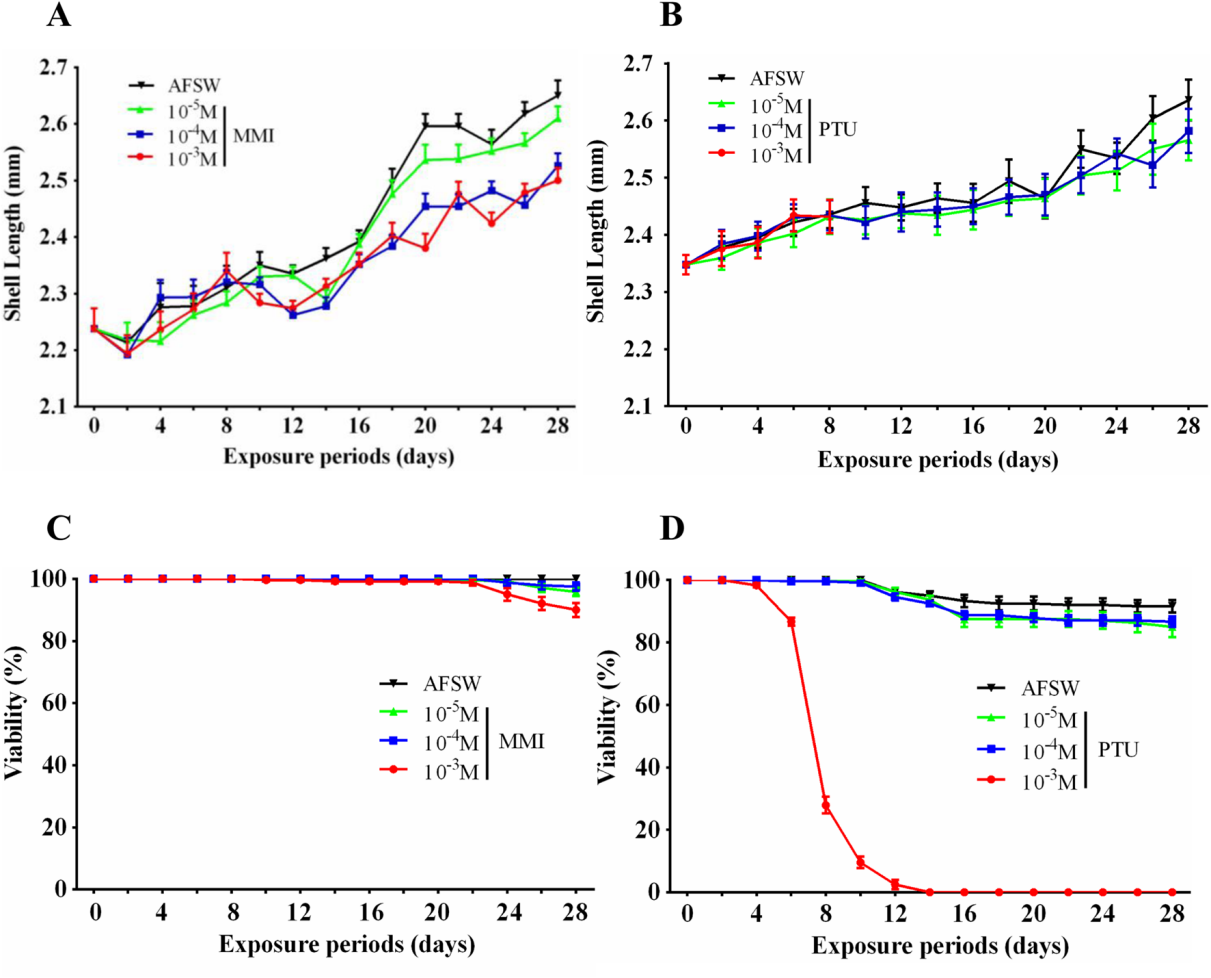
The effect of 4 weeks exposure to MMI and PTU on growth (**A**, **B**) and viability (**C**, **D**) of *M. coruscus* juveniles.

The juveniles of *M. coruscus* were exposed 4 weeks to MMI and PTU for investigating the effect of MMI and PTU on growth (Fig. 6). Shell length of *M. coruscus* juveniles was significantly decreased in 10^–3^ and 10^–4^ M MMI treatment groups compared to the control group after 28 days exposure (*P* < 0.05) (Fig. 6A). 10^–5^ M MMI treatment group had no effect on shell growth relative to control (*P* > 0.05) (Fig. 6A). All concentrations of PTU showed no significant inhibition effect on juvenile growth (*P* > 0.05) (Fig. 6B). Juvenile viability was significantly declined at 10^–3^ M and 10^–5^ M MMI treatment groups compared to the control group after 28 days exposure (*P* < 0.05) (Fig. 6C). Juvenile viability from 6 days onwards significantly declined in the 10^–3^ M PTU treatment group compared to the control group (*P* < 0.05) (Fig. 6D). After exposure for 28 days, 10^–4^ M and 10^–5^ M PTU had no effect on juvenile viability compared to the control group (*P* > 0.05) (Fig. 6D).

## Discussion

Metamorphosis in vertebrates and chordates controlled by thyroid hormone signaling has been well characterized^2^. Notwithstanding all this knowledge, the origins of this ancestral role in non-chordates such as lophotrochozoan are still largely unknown. Here, we have cloned and characterized two deiodinase genes with the TGA codon in the open reading frame and SECIS element in cDNA sequences in *M. coruscus*, indicating two deiodinases were selenoproteins and both containing a highly conserved active catalytic center. The results showed that anti-thyroid compounds MMI and PTU reduced larval metamorphosis in response to the metamorphosis inducer EPI. Surprisingly, lower concentrations had a more pronounced effect than other higher concentrations. The suppression of juvenile growth was observed in higher concentrations of MMI-treated groups after 4 weeks of exposure.

Two deiodinase homologs were cloned in *M. coruscus*. The full-length sequences of McDx and McDy both contain a Sec residue in their active catalytic center and a SECIS element in 3′-UTR. The stem-loop structure of SECIS element identified in 3′-UTR of McDx and McDy is required for the recoding the in-frame UGA to a Sec codon within the coding sequence, thus ensuring the incorporation of Sec into the catalytic center of the protein^28^. The Sec is essential for the catalytic activity of vertebrate deiodinases^30^. The active catalytic center around the Sec codon is highly conserved across invertebrates and vertebrates (Fig. 1). Phylogenetic analysis revealed that deiodinases of molluscs (McDy, CgDx and CgDy) and vertebrates (D2 and D3) shared a node representing an immediate common ancestor, which resembled vertebrates D1 and might suggest that McDy acquired specialized function from vertebrates D1. Furthermore, due to fewer deiodinase sequences reported ahead of Urochordata, three deiodinases from two ascidian species did not cluster with vertebrates deiodinases.

THs controlled metamorphosis is a widespread feature in vertebrates accompany by the morphological and physiological changes observed at the molecular, cellular, and tissue levels^1^. MMI, a goitrogen compound, was applied to block THs production and decreased THs in plasma of vertebrates^12,30,31^. Blocking metamorphosis by MMI-treated tadpoles leads to further growth of body size as well as slight hypertrophy of their thyroid gland, and exposure to exogenous THs can accelerate the metamorphosis of amphibian tadpoles^32,33^. In flatfish, MMI completely blocked metamorphosis, which impaired the migration of their eyes to the same side of the body and remodeling of the head, and demonstrated that THs positively regulated the asymmetric D2 expression in the head tissues^34^. The present study revealed that a significant reduction of larval metamorphosis was only observed after exposure to a lower concentration (10^−5^ M) of MMI, while the high doses of MMI had no effect on larval metamorphosis. PTU is an anti-thyroid drug that selectively inhibits D1-mediated T3 synthesis prescribed for hyperthyroidism in some vertebrates^13^. Furthermore, exposure to PTU impaired zebrafish eye development, visual performance, and swimming activity suppression by inhibiting tyrosine kinase signaling revealed non-TH effects^35,36^. In this study, PTU had a profound effect on inhibiting larval metamorphosis of *M. coruscus*, and the lower concentrations of PTU resulted in a more severe effect than the higher dose. Furthermore, both MMI and PTU did not affect larval viability during the metamorphosis assay (Fig. S2). We observed that metamorphosed larvae in PC groups had the same biological characteristics as MMI and PTU treated groups, including loss of the velum, acquisition of the gills, and post-larval shell growth^24^. The non-metamorphosed larvae in MMI and PTU treated groups all have the velum, and most larvae were swimming, which showed no significant morphological difference compared to PC group. The lower larval metamorphosis rate in MMI and PTU treated groups relative to PC groups revealed fewer pediveliger larvae were competent in response to the inducer EPI (Fig. 5A), suggesting MMI and PTU affected the metamorphic competence of larvae responding to EPI.

Given that the THs are required for the proper function of metamorphosis and development in vertebrates, the unique capacity of the deiodinases is important to modulate THs levels in peripheral tissues in these processes^17^. Previous studies have shown that both MMI and PTU caused a retarded growth performance in tadpoles of *X. laevis*^37^. We found that highly expressed McDx and McDy transcripts in the post-larvae stage, indicating that two deiodinases are important for post-larval development. The function of McDx and McDy require further investigation. We hypothesize that mussels may utilize the iodine from microalgae-derived proteins to iodinate their proteins, and anti-thyroid drugs may affect this process. MMI leads to shell growth retardation in a concentration-dependent manner in juveniles of *M. coruscus* after 4 weeks of exposure, whereas PTU had no effect. It seems to suggest that growth retardation in MMI exposed groups may ascribe to the inhibition of protein iodination in juveniles of *M. coruscus*. Increased dead juveniles in 10^–3^ MMI exposed group of the fourth week imply that iodide bioavailability may be necessary for normal growth. Further studies are needed to understand the iodine accumulated in the mussel tissues. In addition, tyrosine kinase signaling is another way that may also modulate the toxic effects of PTU on larval metamorphosis of *M. coruscus*, which deserves further investigation. Taken together, our data suggest that juvenile growth responded differently to MMI and PTU, even though similar effects on the inhibition of larval metamorphosis were identified. Although THs were found in some molluscs species and can induce larval metamorphosis^6–8^, exposure to THs has no effect on larval metamorphosis of *M. coruscus* (data not shown). What is not clear is if the effect of MMI and PTU on juvenile growth or larval metamorphosis through the iodinated proteins in *M. coruscus*.

In conclusion, two deiodinase genes have been cloned in *M. coruscus* and their active catalytic centers are highly conserved through phylogeny. Our findings suggest that anti-thyroid compounds MMI and PTU significantly reduced larval metamorphosis. The suppression of juvenile growth was only observed in the MMI treatment group in a concentration-dependent manner. Our study provides a clue to uncover the thyroid signaling controlling larval metamorphosis and development of the mussel *M. coruscus*. Further works are required to demonstrate the biochemistry function of McDx and McDy and the crosstalk between iodinated protein in mussel and larval metamorphosis.

## Supplementary Information

Supplementary Figures.

## Data Availability

The datasets generated and analysed during the current study are available from the corresponding author on reasonable request.
