# Supplementary material for: Larval metamorphosis is inhibited by methimazole and propylthiouracil that reveals possible hormonal action in the mussel Mytilus coruscus
Source: Sci Rep. 2021 Sep 29;11:19288. doi: 10.1038/s41598-021-98930-9 (PMC8481496; doi:10.1038/s41598-021-98930-9)
Supplement: Supplementary file 1 — Supplementary Figures. [file 41598_2021_98930_MOESM1_ESM.docx]

**Supplemental Material**

**Larval metamorphosis is inhibited by methimazole and propylthiouracil that reveals possible hormonal action in the mussel *Mytilus coruscus***

Yi-Feng Li^1,2*^, Yu-Qing Wang^1,2^, Yi Zheng^1,2^, Xue Shi^1,2^, Chong Wang^3^, Yu-Lan Cheng^1,2^, Xin Zhu^1,2^, Jin-Long Yang ^1,2^, Xiao Liang ^1,2*^

^1^ International Research Center for Marine Biosciences, Ministry of Science and Technology, Shanghai Ocean University, Shanghai, China

^2^ Key Laboratory of Exploration and Utilization of Aquatic Genetic Resources, Ministry of Education, Shanghai Ocean University, Shanghai, China

^3^ Ocean and Fisheries Research Institute of Binzhou, Binzhou, China.

^*^Correspondence:

Yi-Feng Li, yifengli@shou.edu.cn

Address: College of Fisheries and Life Science, Shanghai Ocean University, 999 Hucheng Huan Road, Shanghai 201306, China

Xiao Liang, x-liang@shou.edu.cn

Address: College of Fisheries and Life Science, Shanghai Ocean University, 999 Hucheng Huan Road, Shanghai 201306, China

**Supplementary figure caption:**


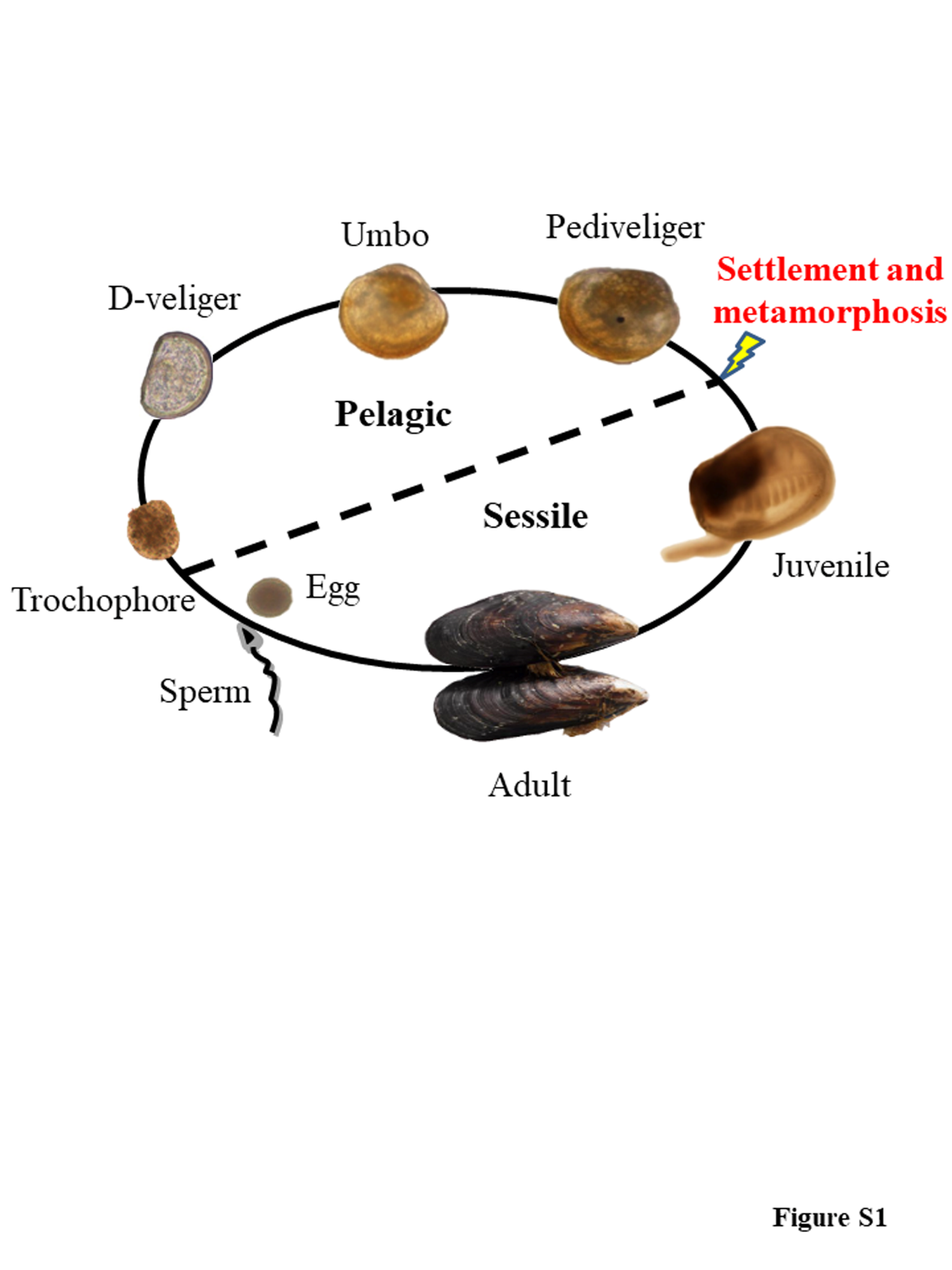


**Figure S1.** The life cycle of *M. coruscus*. Trochophore is a first pelagic larval stage post-fertilization, and swimming D-veliger larvae developed after two days post-fertilization (dpf). The larvae were developed to the umbo and pediveliger stage, which lasts for a month of the planktonic phase prior to settlement and metamorphosis.


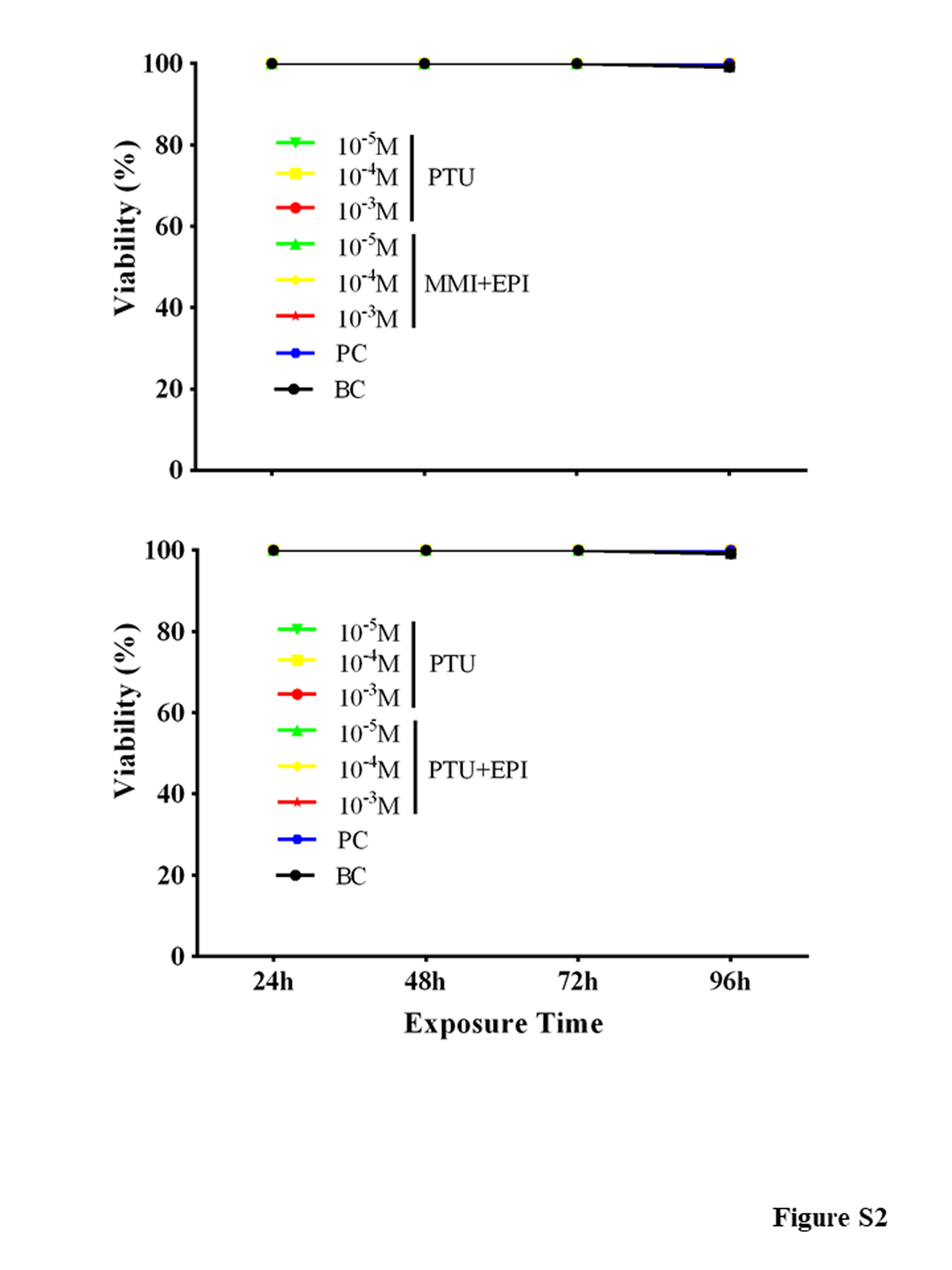


**Figure S2.** Viability of mussel pediveliger larvae in the metamorphosis assay. PC: positive control, 10^-4^ M EPI; BC: Blank control.
